# Winter Habitat Preferences for Florida Manatees and Vulnerability to Cold

**DOI:** 10.1371/journal.pone.0058978

**Published:** 2013-03-20

**Authors:** David W. Laist, Cynthia Taylor, John E. Reynolds

**Affiliations:** 1 Marine Mammal Commission, Bethesda, Maryland, United States of America; 2 Sea to Shore Alliance, Sarasota, Florida, United States of America; 3 Mote Marine Laboratory, Sarasota, Florida, United States of America; The University of Wollongong, Australia

## Abstract

To survive cold winter periods most, if not all, Florida manatees rely on warm-water refuges in the southern two-thirds of the Florida peninsula. Most refuges are either warm-water discharges from power plant and natural springs, or passive thermal basins that temporarily trap relatively warm water for a week or more. Strong fidelity to one or more refuges has created four relatively discrete Florida manatee subpopulations. Using statewide winter counts of manatees from 1999 to 2011, we provide the first attempt to quantify the proportion of animals using the three principal refuge types (power plants, springs, and passive thermal basins) statewide and for each subpopulation. Statewide across all years, 48.5% of all manatees were counted at power plant outfalls, 17.5% at natural springs, and 34.9 % at passive thermal basins or sites with no known warm-water features. Atlantic Coast and Southwest Florida subpopulations comprised 82.2% of all manatees counted (45.6% and 36.6%, respectively) with each subpopulation relying principally on power plants (66.6% and 47.4%, respectively). The upper St. Johns River and Northwest Florida subpopulations comprised 17.8% of all manatees counted with almost all animals relying entirely on springs (99.2% and 88.6% of those subpopulations, respectively). A record high count of 5,076 manatees in January 2010 revealed minimum sizes for the four subpopulations of: 230 manatees in the upper St. Johns River; 2,548 on the Atlantic Coast; 645 in Northwest Florida; and 1,774 in Southwest Florida. Based on a comparison of carcass recovery locations for 713 manatees killed by cold stress between 1999 and 2011 and the distribution of known refuges, it appears that springs offer manatees the best protection against cold stress. Long-term survival of Florida manatees will require improved efforts to enhance and protect manatee access to and use of warm-water springs as power plant outfalls are shut down.

## Introduction

The Florida manatee, *Trichechus manatus latirostris*, is a subspecies of West Indian manatee that occurs almost exclusively in the southeastern United States at the northern limit of the species’ range [1]. Florida manatees, particularly juveniles, are vulnerable to death from cold stress when water temperatures fall below 18–20°C for long periods of time, or to temperatures of 10–12°C or less for periods of a few days or less [2]. Although some areas of Florida (e.g., southeast Florida) are less prone to cold temperatures, even in southernmost Florida water temperatures can fall several degrees below 18°C for a week or more at a time and to 10°C for shorter periods in cold winters [3]. To survive such periods almost all Florida manatees remain near pockets of warm water called “warm-water refuges.” Two functional categories of warm-water refuges have been identified [3], [4]: (1) discharges formed by the constant outflow of warm water mainly from natural springs or power plants, and (2) passive thermal basins (PTBs) heated by solar radiation, ground water seeps, or microbial degradation of benthic organic material. Most PTBs are either deep basins warmed by the sun in the day that cool slowly at night, or basins where warm, salty water is trapped beneath a layer of lighter fresh water from upstream runoff that slows cooling long enough to support animals through brief cold periods [5].

**Figure 1:**
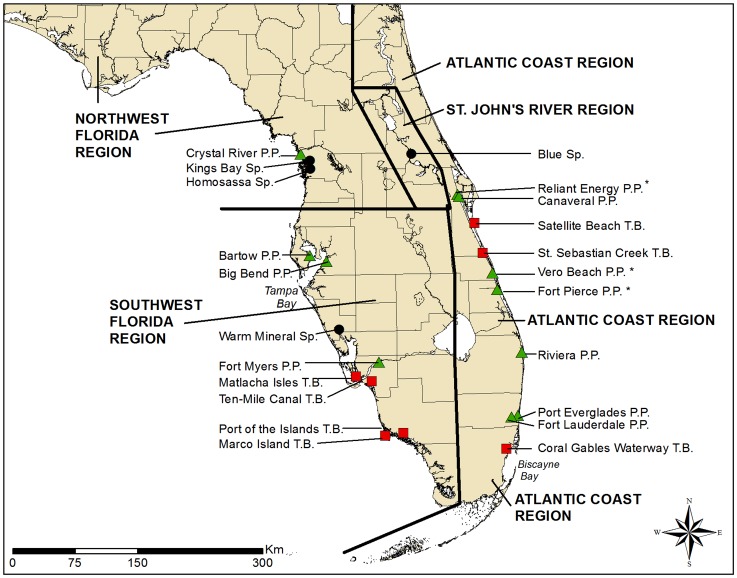
Location of warm-water refuges with counts of more than 50 manatees and boundaries for the four Florida manatee subpopulations. (•  =  springs; ▴ =  power plants; ▪  =  passive thermal basins; *  =  power plants that have been retired, mothballed, or are no longer significant aggregation sites due to reduced operations).

Calves learn to use individual refuges or sets of refuges by following their mothers during the first year of life and typically continue to use those sites as they age [6]. Because of their fidelity to refuges, Florida manatees occur in four relatively discrete regional subpopulations [7], also called management units (Figure 1): (1) the upper St. Johns River, (2) the Atlantic Coast, (3) Northwest Florida, and (4) Southwest Florida. As water temperatures rise in spring, animals disperse from refuges into overlapping ranges along the Atlantic and Gulf of Mexico coasts, but rarely move between coasts [6].

Warm-water refuges used regularly by more than a few animals are well known and many have been subject to annual winter counts since at least the 1980s [7], [8]. The largest manatee aggregations occur at power plant outfalls and springs. All power plant outfalls now used by manatees are at least 35 years old. Although several plants have been or are currently being modernized to extend their operational lives for a few decades (i.e., Ft. Myers plant, Ft. Lauderdale plant, Port Everglades plant (now the Port Everglades Energy Center), Cape Canaveral plant (now the Cape Canaveral Energy Center), and the Riviera plant (now the Riviera Beach Energy Center)), others may be retired within a few years due to outdated technology and high operating costs [3], [4]. Experience with past shut-downs of power plant and industrial outfalls suggests that a potentially large portion of manatees accustomed to using them will remain near those sites rather than move long distances to find a comparable site [3]. Thus, unless another suitable refuge is nearby and known to animals, many are likely to sustain high rates of cold-stress death when plants close [4]. Because of this, eventual plant retirements are recognized as a significant long-term threat to Florida manatees [9], and resource managers, particularly those with the Florida Fish and Wildlife Commission, have begun to protect and enhance selected warm-water refuges to sustain regional subpopulations after power plants are retired.

Refuge temperatures fluctuate to varying degrees depending on refuge type, location, and weather conditions; thus different refuges provide different levels of protection against cold stress. Whereas discharge temperatures at warm-water springs remain nearly constant year-round in all weather conditions, they can fluctuate considerably at power plants. When ambient water temperatures fall to the low teens in extreme cold weather, which is most common in the northern and central thirds of the state, once-through cooling systems at power plants are often unable to elevate discharge temperatures high enough to prevent manatee cold stress. Prolonged cold periods or reductions in freshwater flow also can cause PTBs to cool to potentially lethal temperatures. To classify the ability of different refuges to meet manatee thermal requirements in mild, cold, and severe cold winter conditions, the Florida Fish and Wildlife Commission, U.S. Fish and Wildlife Service, and U.S. Geological Survey recently developed a system to rank refuges into three different categories of reliability (Ronald Mezich, Pers. Comm. 11 September 2011. Wildlife Biologist, Florida Fish and Wildlife Commission, Tallahassee Florida ): (1) high quality refuges (mostly springs) that maintain water temperatures at >22°C in all weather conditions; (2) medium quality refuges (mostly power plants and some PTBs) that remain >22°C in mild winters conditions but can fall to 18°C during severe cold; and (3) low quality refuges (mostly PTBs and power plants that operate intermittently) that remain >20°C in mild winters but have no reliable minimum temperature in severe cold.

Florida manatees are listed as “endangered” under the U.S. Endangered Species Act and Florida state law, but the U.S. Fish and Wildlife Service is considering reclassifying them as threatened [10], [11]. The proposal has been controversial due to uncertainties about (1) when power plants used by manatees will be retired, (2) whether manatees using plants will be able to find alternative refuges, and (3) whether alternative refuges are adequate to support current numbers of manatees after plants close. Although the number of manatee deaths due to plant closures will depend in part on the number of manatees that rely on plant outfalls and the availability of alternative refuges, there has been no systematic attempt to quantify the proportion of animals using different types of refuges either statewide or by regional subpopulation. The closest efforts in this regard are two recent studies to estimate manatee carrying capacity; one estimated warm-water carrying capacity for each of the four regional subpopulations based on expert opinion [12] and the other did so for 11 specific warm-water sites (mostly springs) based on estimates of warm-water area at the site and nearby food resources [13]. To improve assessments of refuge use patterns and the extent to which refuges other than power plants may be able to support regional manatee subpopulations as power plants close, we provide the first attempt to quantify the proportion of Florida manatees using different types of warm-water refuges statewide and regionally. We also evaluate the effectiveness of different refuge types to prevent cold-stress manatee deaths and suggest future management actions.

## Methods

Since 1991 the Florida Fish and Wildlife Research Institute (then the Florida Marine Research Institute) has organized annual state-wide winter counts called “synoptic surveys.” Those surveys attempt to count a maximum number of animals at all regularly used refuges, as well as some other waterways where scattered sightings occur during winter cold fronts when manatees aggregate at refuges in greatest numbers. To avoid double counting, counts are conducted in one or two days with each of Florida’s east and west coasts surveyed in a single day. To evaluate the number of manatees using different warm-water refuge types we examined the distribution and counts for each refuge type during surveys from 1999 to 2011. At least one survey was conducted in every year except 2008, when no survey was conducted due to unusually mild weather. In years when more than one survey was conducted, we used the survey producing the highest count.

Survey data were recorded county-by-county listing the number of manatees counted at known, named refuge locations where at least a few manatees occur every year. Counts at named refuges were made either by aerial survey or ground observers depending on site conditions. Aerial surveys also covered some waterways where experience has shown that scattered animals may occur and as aircraft move between named sites. Sightings away from known refuges where manatee occurrence is unpredictable were recorded by county under a heading called “unnamed sites.” Most sightings away from known refuges involve animals on foraging trips, or in the southern third of the Florida Peninsula, possibly at small unrecognized PTBs or warm-water seeps. For this study, we listed sightings at unnamed sites as “other sites or unknown refuges.” A detailed description of synoptic survey methods is provided on the Florida Fish and Wildlife Commission web site (http://www.myfwc.com/research/manatee/projects/population-monitoring/synoptic-surveys/).

We first sorted annual counts into the four Florida manatee subpopulations (i.e., the upper St. Johns River, Atlantic Coast, Northwest Florida, and Southwest Florida subpopulations) based on regional boundaries identified in the current Florida Manatee Recovery Plan [7] (Figure 1;). We then further divided annual counts at named refuges into one of three types – power plant outfalls, natural springs, or PTBs – based on the warm-water feature known to occur at each site. Thus, all counts classified as power plants were at sites named for a power plant and all counts classified as a natural spring were at sites named for a warm-water spring (e.g., Blue Spring) or small tributary known to have a warm-water spring (e.g., Jenkins Creek, Mud Creek, and Spring Bayou). Sites classified as PTBs were those where manatees aggregate each winter, but where neither power plants nor springs occur (e.g., Ten-Mile Canal, Port of the Islands marina, and Matlacha Isles). All sightings listed as “unnamed sites” were assigned to a catch-all category of “other/unknown” sites recognizing that some could include small unrecognized PTBs or warm-water seeps.

Histograms were prepared for each regional subpopulation showing the number of manatees by year counted at power plants, natural springs, thermal basins, and other/unknown sites. The proportion of manatees at different refuge types in each region was calculated by summing annual counts for each refuge category in a region and dividing it by the total number of sightings in that region across all 13 years of the study period. A statewide assessment was conducted using the same approach for all regions combined in all years.

Between 2 and 13 January 2010 southern Florida experienced its coldest 12-day period since 1940. Temperatures were both unusually cold and persisted for an unusually long period. In Miami air temperatures averaged 11.5°C (52.7° F) and at Tamiami Airport in Miami-Dade County it fell to a low of –3.3°C (26.0°F), the second lowest temperature since records were first kept there in 1948 [14]. In the weeks and months that followed, at least 252 manatees died of acute or chronic cold stress [15]. This was an order of magnitude greater than the average annual number of confirmed cold-stress deaths during the preceding 11 years. To determine if patterns of refuge use differed in that extreme cold period, we examined the 12–15 January 2010 synoptic survey separately following the above methods and compared results for that year to the average distribution over the preceding 11-years.

Manatee preference for different refuge types was also examined by reviewing maximum manatee counts at refuges with at least one winter count of 50 or more animals during either synoptic surveys or any other known counts (e.g., the 30-year record of periodic winter counts at certain Florida Power & Light Company power plants [8]. Those refuges were then grouped for comparison by type (i.e., power plant, natural spring, or PTB) into four categories based on the size of each site’s single highest count: 50–99 manatees, 100–299 manatees, 30–499 manatees, and more than 500 manatees.

Finally, to evaluate how well different refuge types prevented cold-stress deaths, we examined a map showing carcass recovery locations for all manatee deaths attributed to cold-stress from the Florida Manatee Salvage and Necropsy Program from 1999 to 2011. Data on all causes of manatee mortality by year and county are on the Fish and Wildlife Research Institute web site under Florida manatee mortality statistics http://research.myfwc.com/features/default.asp?id=1001. Cold-stress deaths are identified by diagnostic characteristics, such as white patches of necrotic tissue on the skin, white tinged skin on the snout, and depleted fat reserves [2], as well as information on when and where carcasses were recovered. We sorted those deaths by region and examined each region to determine if they accounted for a disproportionately large or small number of cold-stress deaths relative to the proportion of the total manatee population in each region. To estimate what part of the total manatee population was in each region, we followed the approach of the Fish and Wildlife Service [7] using the average proportion of synoptic counts occurring in each region over our 13-year study period. We also visually identified areas with high and low numbers of cold-stress deaths from the map of carcass recovery locations and compared that pattern to types of warm-water refuges known to occur in those areas.

## Results

### Patterns of refuge use

**Figure 2:**
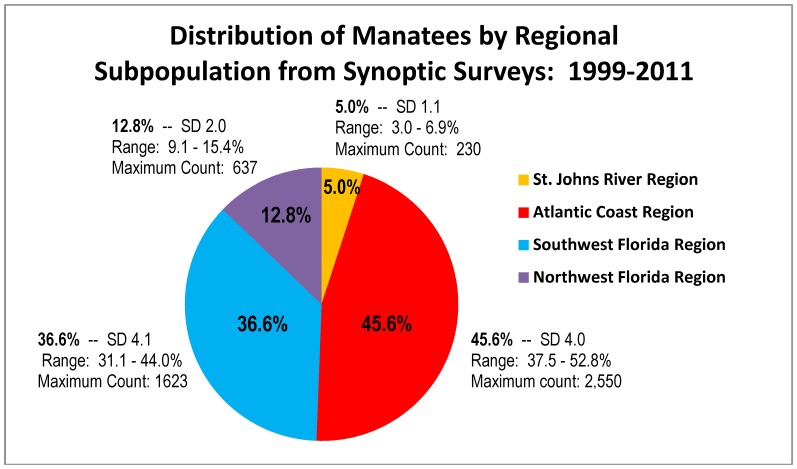
Proportion of manatees and maximum manatee counts (i.e. minimum abundance) for the four regional Florida manatee subpopulations based on synoptic surveys from 1999 to 2011. (SD  =  Standard Deviation; All maximum counts were obtained during the same January 2010 synoptic survey.)

Between 1999 and 2011, synoptic surveys counted 38,058 manatees. The percentage of counts by region over the entire 13-year period with associated standard deviations and ranges of percentages are shown in Figure 2. This distribution is virtually identical to regional proportions reported by the Fish and Wildlife Service from synoptic surveys between 1996 and 2000 – 12%, in Northwest Florida, 37% in Southwest Florida, 47% along the Atlantic Coast, and 4% in the upper St. Johns River [7]. Between 1999 to 2011the maximum count of manatees during a single synoptic survey, which currently is considered the best minimum estimate of total population size at the time of the survey, was 5,076 manatees recorded on 12–15 January 2010. The maximum counts for each of the four subpopulations were also recorded on that survey and are shown on Figure 2.

**Figure 3:**
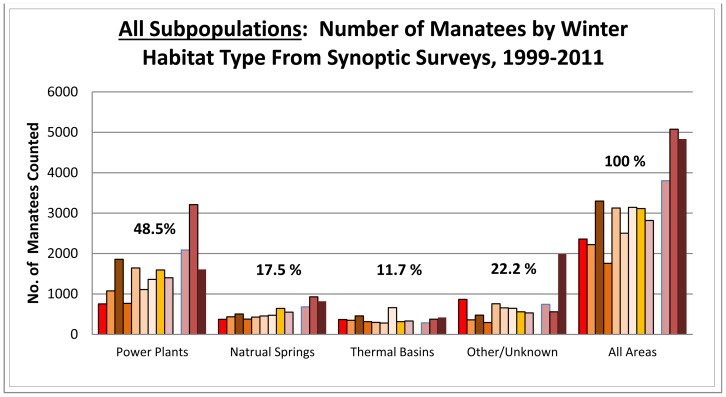
Proportions of manatees at power plants, natural springs, passive thermal basins and other sites during synoptic surveys from 1999 to 2011. (Bars indicate the number of manatees counted each year over the 13-year study period; no survey was conducted in 2008.)

For all areas combined over all years of the study period, 48.5% (SD 9.2, range 31.9–63.2%) of all manatees were counted at power plant outfalls, 17.5% (SD 2.4, range 13.7–21.6%) at natural springs, 11.7% (SD 4.4, range 7.4–21.1%) at PTBs, and 22.2% (SD 8.9, range 11.0–41.1%) at other locations with no known warm-water feature (Figure 3). The strong preference for power plant and spring discharges in years prior to 2010 – 66.0% of all animals (48.5% and 17.5%, respectively) – was even more pronounced during the exceptionally cold January of 2010 when proportional use of power plants and springs increased to 81.6% of all animals (63.2% and 18.3%, respectively). In contrast, the proportion of counts at PTBs and sites with no known warm-water features declined by nearly half from an average of 33.9% of all animals between 1999 and 2009 (13.0% and 20.9%, respectively) to 18.4% (7.4% and 11.0%, respectively) during the exceptionally cold period in January 2010. Most sightings at locations with no known warm-water features were in Florida’s southernmost counties, particularly in Southwest Florida, and likely reflected use of small unrecognized PTBs or warm-water seeps.

Strong preference for power plants was also indicated by their dominance among refuges with the highest counts. There were 19 refuges with maximum winter counts exceeding 50 animals (Table 1, Figure 1). Ten of those were power plant outfalls, including six with counts above 300 manatees. The largest single count at any refuge was also at a power plant (i.e., 957 manatees at the Canaveral power plant in January 2010). Four of the 19 refuges were springs, two of which had counts above 300. The remaining six refuges were PTBs, but only one (Sebastian River) had a count exceeding 300 and that was a single anomalous count of 704 in 2010 that was three times higher than any previous count at that site. Not on this list of major refuges is a diffuse complex of small PTBs in Southwest Florida in the Ten Thousand Islands area of the Everglades, where such sites may support more than 400 manatees in winter [5].

**Table 1:** Maximum counts of manatees (No./Year) at warm-water refuges.

50–99 Manatees	100–299 Manatees	300–500Manatees	>500 Manatees
Power Plants			
Crystal River (63/2007)^ 1^	Reliant Energy (227/2005)^ 1^	Pt Everglades (454/2009)^ 2^	Fort Myers (905/2010)^2^
	Bartow (107/2005)^ 1^	Big Bend (328/2010)^ 1^	Canaveral (957/2010)^ 1^
			Riviera (581/2010)^ 2^
			Ft. Lauderdale (9147/2012)^ 3^
Natural Springs			
	Homosassa Sp. (156/2009)^ 1^	Blue Spring (317/2010)^ 4^	Kings Bay (651/2010)^ 1, 3^
	Warm Mineral Sp. (147/2002)^3^		
Passive Thermal Basins			
Coral Gables C. (62/2005)^ 1^	Port of the Isl. (244/2005)^ 1^		St. Sebastian R. (704/2010)^ 6^
	Matlacha Isles (125/2002)^ 1^		
	10-Mile Canal (121/2005)^ 1^		
	Berkley Canal (140/2011) ^3^		

(Sources: ^1^ Florida Fish and Wildlife Research Institute Synoptic Surveys; ^2^ Reynolds [8]; ^3^ P. Quinn, pers. comm., Broward County Natural Resources Planning and Management Division, Ft. Lauderdale, FL. pers. comm; ^4^Provancha et al. [13]; ^5^ C. Beck, pers. comm. U.S. Geological Survey, Gainesville FL;^ 6^ J. Provoncha, InnoMedicHealth Application, LLC. Merritt Island, FL. pers. comm.).

**Figure 4:**
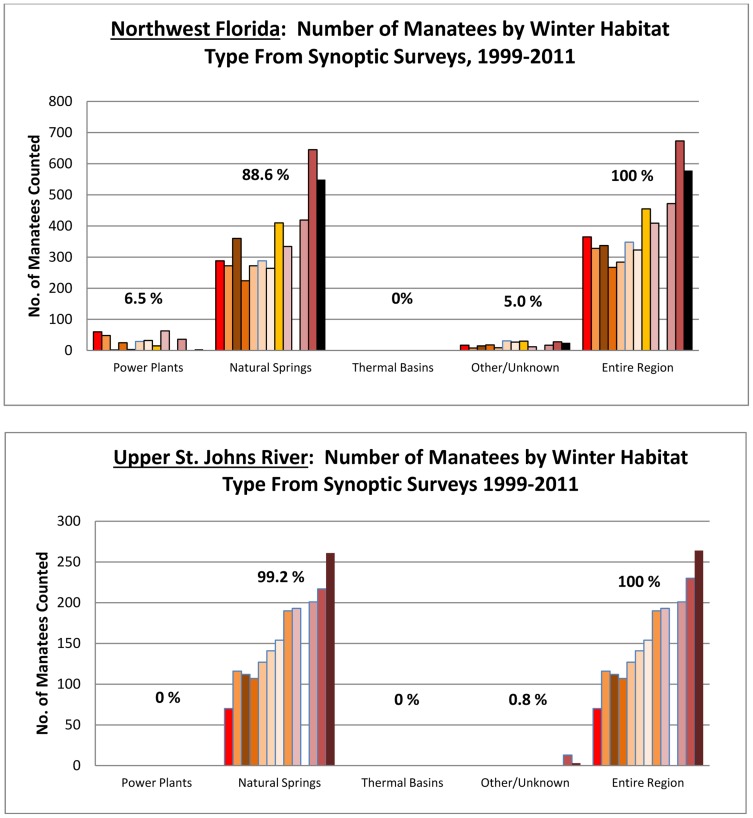
Proportions of manatees in the upper St. Johns River and Northwest Florida subpopulations at power plants, natural springs, thermal basins, and other sites during synoptic surveys from 1999 to 2011. (Bars indicate the number of manatees counted each year over the 13-year study period; no survey was conducted in 2008.)

Interestingly, the two northernmost subpopulations where ambient winter water temperatures tend to be lowest – the upper St. Johns River and the Northwest Florida regions –rely almost exclusively on natural springs (Figure 4). Those regions contain the smallest of the four manatee subpopulations, but their sizes have increased steadily for decades [12], [16]. In the upper St. Johns River almost all manatees counted over the study period (99.1%, 1,889 manatees) were at Blue Spring in Volusia County. Other springs in this region may be in the early stages of hosting overwintering animals. In 2011, 9.5% (25 manatees) were counted at three other regional springs – Salt, Silver Glen, and DeLeon Springs. No power plants or PTBs support manatees in winter in the upper St. John’s River region. In Northwest Florida, 88.6% of all manatees counted (4,325 manatees) were at springs and almost all of those animals were at the spring complex in Kings Bay and at Homosassa Springs. Only 6.5% (317 manatees) were at the one regional power plant used regularly by manatees in winter, and 4.9% (238 manatees) were at other locations with no known warm-water features (i.e., “other/unknown” locations, principally in the Crystal River downstream of the complex of springs in the Kings Bay warm-water refuge).

**Figure 5:**
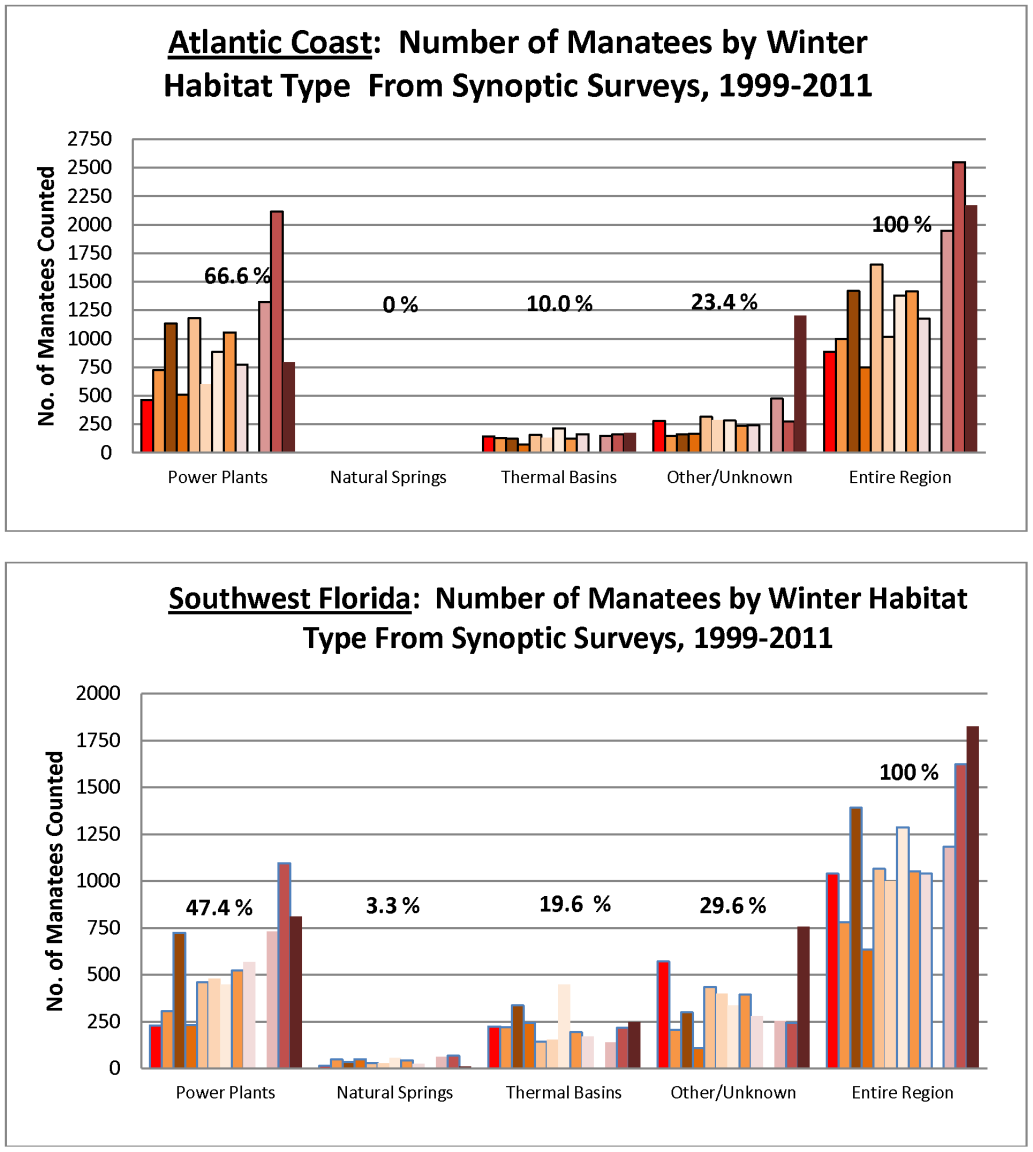
Proportions of manatees in the Atlantic Coast and Southwest Florida subpopulations at power plants, natural springs, thermal basins, and other sites during synoptic surveys from 1999 to 2011. (Bars indicate the number of manatees counted each year over the 13-year study period; no survey was conducted in 2008.)

The two largest subpopulations are along the Atlantic Coast and in Southwest Florida. Together they accounted for 82.2% of all manatees counted. In both regions manatees relied extensively on power plant outfalls and PTBs (Figure 5). Along the Atlantic Coast, where 17,356 manatees were counted over the entire study period, 66.6% (SD 11.9, range52.4–82.9%) were at power plants, 10.0% (SD 3.3, range 6.3–16.3%) at PTBs, and 23.4% (SD 11.9, range 10.7–55.4) at sites with no known warm-water features. No natural springs support manatees along the Atlantic Coast. During the exceptionally cold period in January 2010, the proportion of manatees at power plants was substantially higher than over the previous 11-year average (i.e., 82.9% vs. 68.4%), whereas proportions at PTBs (6.3% vs. 11.3%) and sites with no known warm-water features (10.7% vs. 20.5%) were considerably lower. Most sightings at locations with no known warm-water feature (73.0%) were in the three southernmost counties.

In Southwest Florida counts over the entire study period totaled 13,917 manatees; 47.4% (SD 12.4, range 21.1–67.5%) at power plants, 19.6% (SD 9.2, range11.7 –38.5%) at PTBs, 29.6% (SD 11.5, range 15.0–55.0%) at locations with no known warm-water feature, and 3.3% (SD 2.1, range 0.5–7.7%) at springs. Almost all manatees at springs were at the region’s only large spring, Warm Mineral Spring. Like the Atlantic Coast region, a large majority (90.0%) of manatees in Southwest Florida were at locations with no known warm-water features in the three southernmost counties. Also like the Atlantic Coast region, during the exceptional cold in January 2010, the proportion of manatees at power plants increased substantially compared to the previous 11-year average ( from 44.9 to 67.5%), but decreased at PTBs (from 21.7 to 13.3%) and sites with no known warm-water features (from 31.3 to 15.0%).

### Effectiveness of Refuge Types

**Figure 6:**
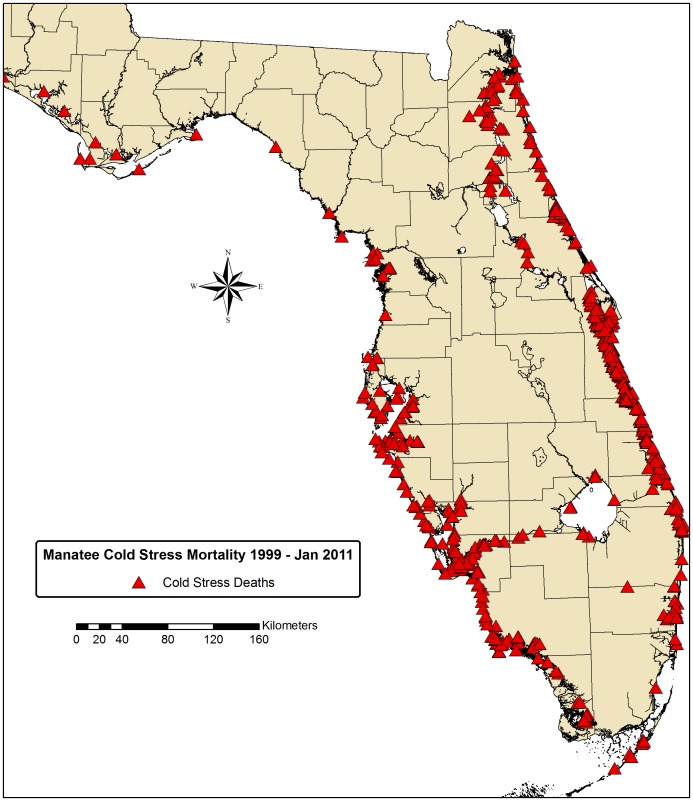
Location of manatee deaths attributed to cold stress, 1999–2011.

From 1999 through 2011, 713 manatee deaths were attributed to cold stress (Figure 6). Over half were recovered in 2010 (252 deaths) and 2011 (113 deaths). The two subpopulations most dependent on springs (i.e., the upper St. Johns River and Northwest Florida) accounted for 6.3% of all cold-stress deaths, which was disproportionately lower than their combined estimate of 17.8% of the total statewide living manatee population. The upper St. Johns River subpopulation representing 5% of all living Florida manatees accounted for only 2.5% (n  =  18) of all cold-stress deaths; the Northwest Florida subpopulation comprising 12.8% of all living Florida manatees accounted for just 4.6% (n  =  33) of all cold-stress deaths. In both regions most deaths due to cold occurred in northern areas farthest from the principal warm-water springs; 15 of the 33 deaths in Northwest Florida were in the Florida Panhandle and 13 of the 18 deaths in the upper St. Johns River were in Putnam County near the northern border with the Atlantic Coast region.

The greatest number of cold-stress deaths was in the Atlantic Coast region where most manatees rely on power plants. That region accounted for a disproportionately high number of cold-stress deaths (55.3%; n  =  394) compared to its estimated proportion (45.6%) of all living Florida manatees. The proportion of deaths due to cold in Southwest Florida, where

manatees rely mostly on a combination of power plants and PTBs, was intermediate between the two northernmost regions and the Atlantic Coast. It sustained 37.3% (n  =  266) of all confirmed cold-stress deaths, roughly equal to its estimated complement (35.6%) of the entire Florida manatee population. However, the Southwest Florida region includes remote parts of the Everglades where carcass recovery is difficult. As a result, carcasses throughout much of that region – particularly the Ten Thousand Islands area – were verified by airplane but often went unrecovered or were recovered too decomposed to determine a cause of death. During the exceptionally cold year of 2010 when 125 carcasses were recorded in this region, only 27 were recovered in a condition that allowed a determination of cause of death, and 24 of those were attributed to cold stress. A large number of the remaining unrecovered (n  =  55) and badly decomposed (n  =  39) carcasses were suspected cold-stress victims [14]. If suspected cold-related deaths are considered, the Southwest Florida region would have sustained a disproportionately large number of cold-stress deaths similar to the Atlantic Coast region.

The geographic pattern of cold-stress deaths revealed that most occurred in two areas (Figure 6). One was a 200-kilometer stretch along the middle third of the Atlantic Coast region (i.e., Brevard, Indian River, and St. Lucie counties) where 35.5% (n = 253) of all cold-stress deaths were recovered. This area marks the northern half of the area used by most of the region’s overwintering animals. Power plants and a few PTBs provide this area’s only warm-water refuges. The other area with high numbers of cold-stress deaths was the southern part of the Southwest Florida region, where 25.4% (n = 181) of all such deaths were recovered and where high numbers of suspected but unverified cold-stress deaths also occurred (i.e., Lee, Collier, and Monroe counties). Power plants and PTBs also provide the only refuges in that area. Interestingly, the distribution of cold-stress-deaths also reveals very low numbers of cold-related deaths in the southernmost 225 kilometers of the Atlantic coast region, even though it lies at the same latitude where high numbers of cold-stress deaths were concentrated in Southwest Florida (Figure 6). The southernmost 225 kilometers of the Atlantic Coast (i.e., Broward, Miami-Dade, and Monroe Counties) accounted for just 3.4% (n  =  24) of all confirmed cold-stress deaths.

## Discussion

Synoptic survey counts confirm that the two smallest subpopulations of Florida manatees – the upper St. Johns and Northwest Florida subpopulations – rely primarily on warm-water springs with little or no use of power plant discharges or PTBs. Conversely, the two largest subpopulations – the Atlantic Coast and Southwest Florida subpopulations – rely largely on power plants and PTBs. Overall, power plants may be used by half of all Florida manatees on the coldest winter days, including up to two-thirds of all animals along the Atlantic coast and nearly half of all manatees in Southwest Florida. Natural springs and PTBs, each of which now supports perhaps 20 to 25% of all manatees, will become virtually the only refuge types for manatees after power plants with outfalls now used by manatees are retired.

These distributions need to be considered with some caution given limitations of synoptic survey methodology. Chief among them is uncertainty in the number of manatees not counted during surveys. It has not yet been possible to develop reliable correction factors to account for animals not seen at most refuges due to inconsistencies in water clarity and weather conditions, and animals that may be away from known refuges at the time of any given survey. For these reasons, synoptic survey data have not been used to estimate total population size or short-term trends in abundance [17]. Nevertheless, synoptic survey data have been considered the best information on overall population size and winter distribution. For example, the U.S. Fish and Wildlife Service used synoptic survey results from 1996 and 2000 to estimate the proportion of the total population in each of the subspecies’ four regional subpopulations [7], and the core biological model used to assess the status and future trends relies on maximum counts from synoptic surveys for minimum estimates of population size [16]. Thus, until better information becomes available on the number of animals not counted in synoptic surveys [17], those who must plan for long-term management of essential refuge habitat should consider synoptic survey results using appropriate caution.

Synoptic surveys likely capture manatee use of springs and power plants most completely. The number of those refuges used by significant numbers of manatees is relatively small and well known. Although some springs used by small numbers of manatees or used only occasionally are not included in surveys, once a few animals begin using them regularly, they have been added to the list of survey sites. For example, in 2011, counts were made for the first time at three springs on the upper St. Johns River – Salt, Silver Glen, and DeLeon Springs – yielding counts of 16, 3, and 6 manatees, respectively. Similarly, Wakulla Spring in Northwest Florida was added to the 2011 survey producing a count of 37 manatees. Because virtually all springs potentially suitable for manatees are visited regularly by people, they are likely to become known to researchers before large numbers of manatees begin to use them. Information about the location of power plant outfalls is even better known. Because power plant operators must monitor their outfalls closely for a variety of reasons, it is very doubtful there are any power plants being used by large numbers manatees that are not known. The refuge type likely to be most underrepresented in this analysis is PTBs. Many unknown sites used by a few manatees may be scattered widely across remote, hard to survey mangrove swamps and the tens of thousands of kilometers of dredged boat canals in southern Florida. Cumulatively, those sites (which could include many of the unnamed sites recorded in synoptic surveys) may be far more significant than indicated in this review.

Caution also is needed with regard to data on the locations where cold-stress deaths are recovered. Although some animals die quickly of acute cold stress and are likely found in the area in the area in which they were first exposed to cold, others that die of chronic cold-stress impacts over a period of weeks or months may move great distances. However, because of refuge site-fidelity patterns and behavioral lethargy typically associated with cold stress [2], we believe the overall pattern of cold-stress carcass recovery locations likely is a useful first order approximation of areas where exposure to cold stress occurred.

Results of this study indicate that natural springs offer the best protection against cold stress, whereas passive thermal basins provide only moderate protection even in southernmost Florida, and power plants provide highly variable levels of protection depending largely on where they are located and whether they operate intermittently. Before power plants were built in the mid-1900s, the best information on the northern limits of manatees in winter is from studies by Moore [18], who suggested Sebastian Inlet on the Atlantic Coast and Charlotte Harbor on the Gulf Coast marked the species northern limit in winter. Manatee use of springs farther north had likely been eliminated by earlier hunting [3]. Power plants have effectively extended the range of the Southwest Florida and Atlantic Coast subpopulations farther north and made formerly unavailable foraging grounds available to overwintering manatees. If all power plants now used by manatees are retired over the next 40 to 50 years and no further steps are taken to prepare for effects of those closures well in advance, strong regional site-fidelity patterns would make it highly unlikely that manatees would move to springs in other regions. Some manatees along the Atlantic Coast are likely to move farther south to PTBs based on tagged movements of some overwintering animals in Brevard County [6]; however, many manatees – particularly those relying on power plants near or north of Sebastian Inlet on the Atlantic Coast and Charlotte Harbor in Southwest Florida – are likely to die of cold-stress as their preferred outfalls disappear [3], [4].

As indicated by findings in this analysis, even in the southern half of the Southwest Florida region where PTBs are almost the only available refuge type other than power plants, high levels of cold stress can occur in severe winters. In the southernmost parts of the Atlantic Coast region, manatees seem less vulnerable to cold stress, possibly because of moderating influences of the Gulf Stream on the area’s ambient water temperatures. Recent observations of manatees in ocean waters off Palm Beach on cold winter days (Reynolds, unpublished data) may suggest some manatees take advantage of ocean waters warmed by the Gulf Stream. It also is possible, however, that low levels of cold stress in the southern part of the Atlantic Coast region are due to major power plants whose locations at lower latitudes enable their outfalls to remain more consistently above 18-20° C. Nevertheless, some analyses of effects of power plant closures on the Atlantic Coast and Southwest Florida subpopulations have suggested a possible 30 to 50% decline in manatee abundance after power plants close due to the associated loss of warm-water carrying capacity [12].

The best opportunity to compensate for potential declines in Atlantic Coast and Southwest Florida subpopulations due to power plant closures is growth of the two subpopulations dependent on springs – the upper St. Johns River and Northwest Florida – before power plants are retired. The three springs now used by most manatees (i.e., Crystal River Springs Complex, Homosassa Springs, and Blue Spring) are first-order magnitude artesian springs (i.e., discharges >100 ft^3^/s; 2.83 m^3^/s) with discharge water temperatures remaining nearly constant at 22°C (± a few tenths of a degree) [19]. Twenty first-order magnitude springs discharge water at temperatures of 22°C or higher in Florida; all of which are in the central or northern parts of the state and most of which are along the Suwannee and the St. Johns River or their tributaries [3], [19]. Almost all of the state’s second-order magnitude springs (i.e., discharges of 10-100 ft^3^/s; 0.283 - 2.83 m^3^/s) also occur in those regions.

### Management Implications

Recent decisions by Florida Power & Light Company to modernize three Atlantic Coast power plants (i.e., the Canaveral, Riviera, and Port Everglades plants) and past modernization of another plant (i.e., the Fort Lauderdale plant), make it unlikely power plant closures will sustain manatees along the Atlantic Coast over the next 30 to 40 years. In southwest and central Florida, three plants have been modernized (i.e., the Fort Myers, Barstow, and Bayside plants), but the primary plant in Tampa Bay, the TECO Big Bend Plant, has not been modernized and could be retired within five to ten years and significantly affect that region’s subpopulation.

Federal and state management agencies are not in favor of costly high-maintenance technological refuges (e.g., gas fired water heaters) to replace power plant outfalls on a long-term basis [20]. Thus, natural springs and PTBs likely will become the primary types of warm- water refuges. With warm-water springs offering the best natural protection against cold stress, we suggest that the most important actions to be taken before power plant outfalls are eliminated are steps to promote greater manatee access to and use of natural springs in central and northern Florida.

Development over the past century has significantly impeded manatee use of natural springs in Florida. Manatee access to some springs – as well as important river habitat –has been blocked by dams. Other springs have been encircled with concrete structures to create private or public swimming holes. Still others have become clogged with silt from development and public use making spring runs too shallow for manatees to navigate, or are exposed to intensive public use that deters manatee use of warm-water areas [21]. To mitigate effects of inevitable power plant closures, a long-term program to improve manatee access and protection at springs is required. The Florida Fish and Wildlife Conservation Commission in particular has begun taking important, well-placed steps in this regard, but further actions are needed. Among them are the following: (1) federal and state acquisition of springs now in private hands for uses that include manatee habitat; (2) removal of dams obstructing manatee access to major springs and river segments, particularly those along the Ocklawaha and Withlacoochee Rivers; (3) restoration of structurally modified springs to more natural conditions; (4) restoring former depths to spring runs that have become too shallow for manatees; (5) improving measures to limit human activities that disrupt manatee use of springs during the winter season; and (6) experimental efforts to move some manatees (perhaps initially using rescued animals scheduled for release back into the wild) from the Atlantic Coast and Southwest Florida regions to springs now unused or little used by manatees (e.g., Silver Spring on the Ocklawaha River and Rainbow Spring on the Withlacoochee River). Such work should be guided by an effort to identify long-term networks of springs and PTBs in each region emphasizing actions that are most feasible and most likely to support significant numbers of manatees.

Along the Atlantic Coast and in Southwest Florida where springs are absent or rare, steps could be explored to test options for creating new warm-water discharges by drilling new wells or opening existing wells to release warm water from saltwater aquifers to create new refuges in small embayments. This could be particularly helpful in southern parts of the two regions to supplement PTBs that may not be adequate to support all manatees in severe winters.

It has taken more than 50 years to create today’s high level of manatee reliance on power plant outfalls for warmth. By the same token, it will likely require a comparable span of decades to assess, identify, and implement measures to restore and establish reliable regional networks of natural springs and passive thermal basins to compensate for likely manatee losses when plants close. What must not occur is postponing needed work until plants close.

It is also vital for the key “players” to work together as a team to accomplish critical goals and objectives. We believe Florida electric utilities, along with the responsible state and federal agencies, bear responsibility for funding actions to support manatees after power plants close. There are likely a number of possible ways by which utilities, agencies, and concerned conservation groups can cooperate to achieve important goals; as an example, to fund actions needed to restore or enhance networks of warm-water refuges in advance of power plant closures, management agencies could require Florida power companies to make annual contributions to a revolving fund that agencies could draw on to cover the costs of actions such as those noted above. Another possibility suggested by a public utility representative many years ago involved acquiring permission from the Public Service Commission to allow utilities to charge a small amount more for electricity and gas, with the stipulation that the funds raised be deposited into a mitigation fund dedicated to conservation and management issues associated with power generation and effects of plant closures.

When the Fish and Wildlife Service considers downlisting Florida manatees from endangered to threatened under the Endangered Species Act, as it is poised to do, it must consider not only their current abundance and trends, but also the extent to which threats to manatees and their habitats are understood and under control. If left unmitigated in the near future, reduction in the number of power plant outfalls on which large numbers of animals now depend may be the greatest threat to manatees over the next 50 years as plants are closed and replaced with new facilities that cannot be permitted to construct comparable outfalls.

To mitigate foreseeable losses of power plant outfalls, an increasing proportion of manatees will need to rely entirely on natural springs and PTBs. Like the increasing number and intensity of hurricanes in Florida, the exceptionally cold winters of 2010 and 2011 may be an example extreme weather that could occur more frequently in Florida as a result of climate change. This would increase cold-stress risks for Florida manatees. Because PTBs provide limited protection against cold stress, preventing a significant decline in manatee numbers as power plants are retired will require a significant increase in the proportion of manatees relying on natural springs for warm-water refuges. The proportions of manatees using different refuge types reported in this paper provide a basis for assessing the scale of the threat posed by eventual plant closures and a baseline against which to measure progress towards mitigating long-term risks. Results provided here suggest there has been virtually no increase in the proportion of manatees using springs over the past decade. As the Fish and Wildlife Service evaluates downlisting options, it should consider the proportion of manatees likely to be affected by eventual power plant closures, the time and resources needed to implement measures to promote manatee use of alternative warm-water refuges to compensate for inevitable power plant retirements, and the adequacy of measures in place to assure that those mitigation measures will be carried out in a timely manner over the coming decades well before plant retirements.

Whatever the ultimate specific solutions may be, it is clear that those solutions require cooperation by multiple partners, implementation in the near future, and a long-term commitment for their support. Without proactive management in advanced of power plant retirements, it is inevitable that warm-water carrying capacity for manatees will change dramatically over the next several decades. The actions we propose above provide some guidance for decision makers and are achievable within that time frame. Without these steps, we believe that much of the gain in the manatee population over the past 40 years could be lost quickly.
